# Use of Oral Antivirals Ritonavir-Nirmatrelvir and Molnupiravir in Patients with Multiple Myeloma Is Associated with Low Rates of Severe COVID-19: A Single-Center, Prospective Study

**DOI:** 10.3390/v15030704

**Published:** 2023-03-08

**Authors:** Vassiliki Spiliopoulou, Ioannis Ntanasis-Stathopoulos, Panagiotis Malandrakis, Maria Gavriatopoulou, Foteini Theodorakakou, Despina Fotiou, Magdalini Migkou, Maria Roussou, Evangelos Eleutherakis-Papaiakovou, Efstathios Kastritis, Meletios A. Dimopoulos, Evangelos Terpos

**Affiliations:** Department of Clinical Therapeutics, School of Medicine, National and Kapodistrian University of Athens, 11528 Athens, Greece

**Keywords:** multiple myeloma, molnupiravir, ritonavir-nirmatrelvir, SARS-CoV-2, belantamab mafodotin

## Abstract

In patients with multiple myeloma (MM), SARS-CoV-2 infection has been associated with a severe clinical course and high mortality rates due to the concomitant disease- and treatment-related immunosuppression. Specific antiviral treatment involves viral replication control with monoclonal antibodies and antivirals, including molnupiravir and the ritonavir-boosted nirmatrelvir. This prospective study investigated the effect of these two agents on SARS-CoV-2 infection severity and mortality in patients with MM. Patients received either ritonavir-nirmatrelvir or molnupiravir. Baseline demographic and clinical characteristics, as well as levels of neutralizing antibodies (NAbs), were compared. A total of 139 patients was treated with ritonavir-nirmatrelvir while the remaining 30 patients were treated with molnupiravir. In total, 149 patients (88.2%) had a mild infection, 15 (8.9%) had a moderate infection, and five (3%) had severe COVID-19. No differences in the severity of COVID-19-related outcomes were observed between the two antivirals. Patients with severe disease had lower neutralizing antibody levels before the COVID-19 infection compared to patients with mild disease (*p* = 0.04). Regarding treatment, it was observed that patients receiving belantamab mafodotin had a higher risk of severe COVID-19 (*p* < 0.001) in the univariate analysis. In conclusion, ritonavir-nirmatrelvir and molnupiravirmay prevent severe disease in MM patients with SARS-CoV-2 infection. This prospective study indicated the comparable effects of the two treatment options, providing an insight for further research in preventing severe COVID-19 in patients with hematologic malignancies.

## 1. Introduction

Severe SARS-CoV-2 infection (Coronavirus disease 2019, COVID-19) is characterized by an initial viral phase, often followed by a severe inflammatory phase. In patients with MM, SARS-CoV-2 infection has been associated with a severe clinical course and high mortality rates, due to the concomitant disease- and treatment-related immunosuppression [1]. Furthermore, MM patients respond poorly to vaccination despite adequate immunization, especially those on treatment with anti-CD38 or anti-BCMA therapies [2,3,4,5]. Thus, MM patients are at a higher risk for breakthrough infection compared with noncancer patients or patients with solid tumor, and supportive measures along with prophylactic use of monoclonal antibodies against SARS-CoV-2 are needed [6,7,8]. At the time of SARS-CoV-2 infection, the use of antiviral therapy may improve patient outcomes [9]. Specific antiviral treatment involves viral replication control with monoclonal antibodies and antivirals, including molnupiravir and the ritonavir-boosted nirmatrelvir [10]. Both molnupiravir and ritonavir/nirmatrelvir have shown to reduce the risk for severe COVID-19, including hospitalization and/or death, compared with placebo in unvaccinated individuals [11,12]. More recent data including vaccinated patients with mild COVID-19 support the early administration of antivirals for preventing severe outcomes [13]. Although available evidence supports the use of antivirals in patients with SARS-CoV-2 infection to prevent severe disease, relevant data on MM patients are scarce. This prospective study investigated the effect of these two agents on SARS-CoV-2 infection severity and mortality in patients with MM.

## 2. Materials and Methods

Consecutive patients with MM and SARS-CoV-2 infection were prospectively enrolled in the study from February 2022 to October 2022. During this period, the Omicron SARS-CoV-2 variant, including BA.2, BA.4, BA.5, and BQ.1, was dominant in the country. All patients had microbiologically confirmed SARS-CoV-2 with polymerase chain reaction (PCR). The patients received either ritonavir-nirmatrelvir or molnupiravir according to the national guidelines and the availability of each drug. Treatment with antivirals was initiated within the first five days from COVID-19 symptoms onset in all patients with no need of supplemental oxygen. All patients were at high risk for severe COVID-19 due to the underlying MM. There were no special characteristics determining which drug was given to each patient. Baseline demographic and clinical characteristics, as well as levels of neutralizing antibodies (NAbs), were collected and compared. The effect of different treatments on SARS-CoV-2 infection severity and mortality was examined. The study was approved by the institutional review board.

Neutralizing antibodies (NAbs) against SARS-CoV-2 were determined using an FDA approved methodology (enzyme-linked immunosorbent assay, cPass SARS-CoV-2 NAbs Detection Kit; GenScript, Piscataway, NJ, USA). Anti-SARS-CoV-2 neutralizing antibodies are of particular importance because they inhibit the binding of the receptor-binding domain (RBD) of the surface spike (S) protein to the human angiotensin-converting enzyme 2 (ACE2) receptor [14].

Statistical analysis: Frequencies and percentages were used to describe categorical data, while means and standard deviations were used to describe scale measurements. The Fisher’s exact test was applied to examine the differences in the outcome based on the drug [15]. After applying the Shapiro–Wilk criterion for normality assessment, analysis of variance was applied to examine differences in NAbs count, followed by multiple comparisons under the Tukey’s HSD criterion. A binary logistic-regression model was applied to define ORs of the variables examined, instead of a multinomial logistic-regression model, as the treatment with belantamab was not recorded in patients with a moderate outcome. The significance level was set at 0.05 in all cases and the analysis was carried out with the SPSS v 26.0 software.

## 3. Results

A total of 169 patients infected with SARS-CoV-2 were included. Of those, 74 were female, the average age was 64.4 years, and the mean body mass index (BMI) was 26.91 kg/m^2^. Table 1 shows the baseline characteristics of included patients before SARS-CoV-2 infection. The performance status (PS) is described as the status of symptoms and functions with respect to ambulatory status and need for care. The majority of the patients (78.7%) were in PS 0, which indicates normal activity, and the other 21.3% were in PS 1, which indicates some symptoms but that the patient is still ambulatory. Regarding the medical history, 14 patients (8.3%) were diagnosed with diabetes mellitus (DM), 71 (42%) had hypertension, six (3.6%) had coronary artery disease (CAD), and 16 (9.5%) had chronic obstructive pulmonary disease (COPD).

All patients, except for one, were vaccinated, mostly with the BNT162b2Pfizer/BioNTech vaccine (96.4%). A total of 153 patients had already received three doses, 7 had received four doses, and 8 had received two vaccine shots before SARS-CoV-2 diagnosis. The median time of the last vaccine dose to COVID-19 diagnosis was 6 months (range 1–11 months). No other pharmacological intervention was adopted as prophylaxis against SARS-CoV-2. NAbs levels were not significantly different according to MM stage or line of treatment. Only patients under anti-BCMA therapy had significantly lower NAbs titers compared to the others (*p* < 0.05).

Regarding treatment for symptomatic MM, several drug combinations were administered to patients depending on their medical condition and history of the disease. These included regimens based on proteasome inhibitors, immunomodulatory drugs, anti-CD38 monoclonal antibodies, anti-BCMA treatments (belantamab mafodotin), and other treatments including selinexor and cyclophosphamide (Table 2). For most of the patients (n = 100) this was their 1st line of treatment, for 36 it was their 2nd, for 14 their 3rd, for 7 their 4th, and for 3 patients their 5th line of treatment. A total of 138 patients (81.7%) was receiving dexamethasone as part of their treatment for MM.

As far as the administration of SARS-CoV-2 antivirals is concerned, 139 patients (82.2%) were treated with ritonavir-nirmatrelvir, while the remaining 30 patients (17.8%) were treated with molnupiravir. The duration of antiviral treatment was equal to five days in all but three cases. These three patients were hospitalized before the completion of the antiviral regimen and treatment was interrupted. Antivirals were well tolerated and no major adverse events were noted. Diarrhea grade 1 was reported in 10 patients (6%). All 10 patients were receiving ritonavir-nirmatrelvir. Regarding COVID-19 outcomes as defined by the WHO, 149 (88.2%) patients had mild infection with several signs and symptoms, such as fever, cough, and headache, but not shortness of breath or dyspnea. Fifteen (8.9%) patients experienced moderate infection with evidence of lower respiratory disease during clinical assessment or imaging. Five (3%) patients developed severe COVID-19 and required oxygen support, with complications such as respiratory failure, acute respiratory distress syndrome, sepsis and septic shock, thromboembolism, and/or multi-organ failure.

Table 3 shows that severe cases were treated differently, as expected, compared to mild or moderate cases. An exception was observed regarding the use of corticosteroids for COVID-19, since it appears that they were given almost equally to patients with moderate and severe outcomes.

Table 4 shows that no differences in the severity of COVID-19 outcomes were observed between the two antivirals (*p* = 0.236). COVID-19 infection was resolved in all patients, except for three fatal cases. No differences were observed in the baseline characteristics among patients who received molnupiravir and ritonavir-nirmatrelvir.

Table 5 shows the distribution of gender and myeloma stage according to the outcome of COVID-19 infection. Gender and myeloma stage according to the revised international staging system for myeloma (R-ISS) did not seem to differentiate patient outcomes.

As shown in Table 6, no significant differences were observed in the infection outcomes according to BMI, age, or past medical history defined as the presence of DM, COPD, CAD, or hypertension.

**Figure 1:**
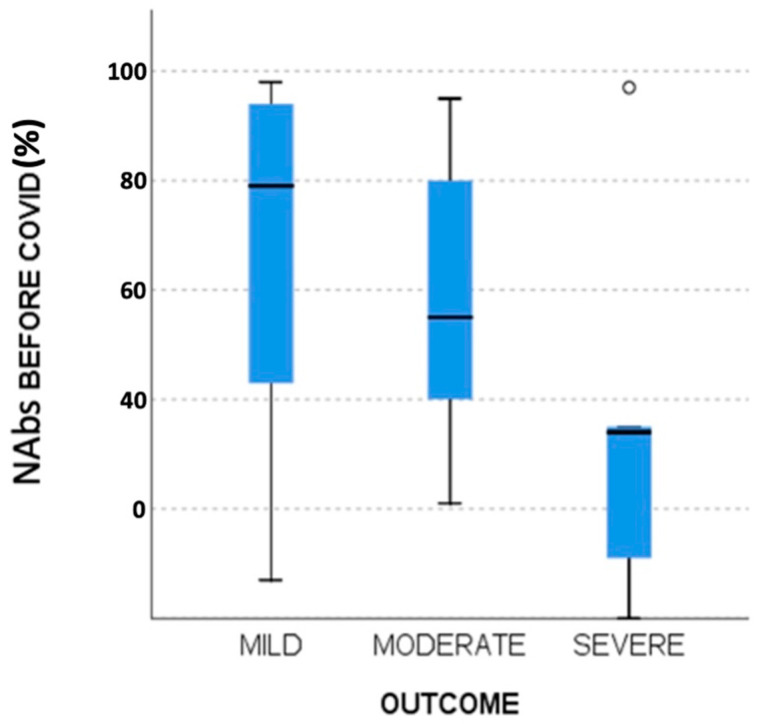
Boxplot of the differences in NAbs before SARS-CoV-2 infection based on COVID-19 outcomes.

An initial approach to differences in patient outcomes showed that regarding PS, a statistically significant difference of mild versus severe cases (*p* = 0.041) was observed, while the difference between mild and moderate cases was borderline non-significant (*p* = 0.052). A difference between severe and mild cases was observed concerning NAbs response levels before SARS-CoV-2 infection (Table 7). As shown in Figure 1, patients with severe disease appeared to have lower NAbs levels before SARS-CoV-2 infection (median time from last NAbs measurement to infection date: 16 days; range: 8–24 days) compared to patients with mild disease (median: 18 days; range 4–27 days, *p* = 0.04). The mean and standard deviations for NAbs levels were 67.49% ± 28.85%, 59% ± 24.69% and 35.4% ± 37.57% for the groups of mild, moderate, and severe outcomes, respectively. Only four patients (2.4%) had lower than 10% neutralizing antibody levels before COVID-19 diagnosis, and two of them experienced a severe infection.

**Figure 2:**
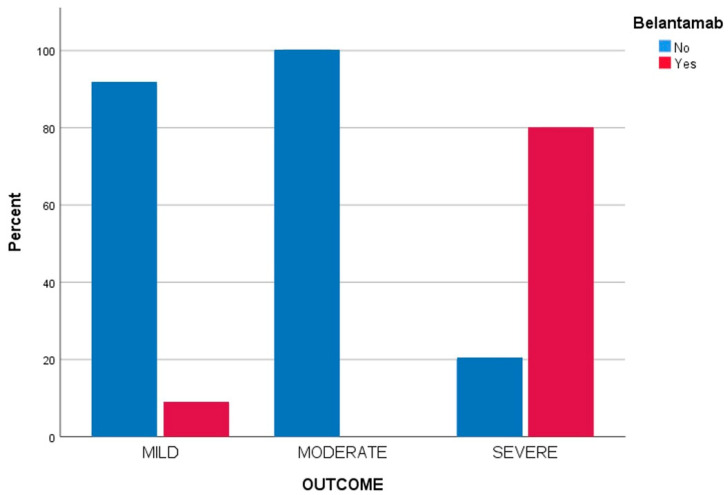
Bar chart of the differences in COVID-19 outcomes according to type of active treatment.

Regarding treatments, despite the various drug types included in the study and their combinations, it was observed that treatment with belantamab mafodotin was associated with adverse COVID-19 outcomes. A total of 47% of patients receiving belantamab mafodotin were male, with a median age of 66.2 years, and a median Nab measurement before the infection of 29.8%. As far as their response to treatment for MM was concerned, five patients had a partial response to therapy (29.4%), five patients had a very good partial response (29.4%), and seven patients had a complete response (41.2%). Seven of these patients received molnupiravir (41.2%) and ten received ritonavir-nirmatrelvir (58.8%). As shown in Figure 2, there was a significantly higher risk for severe SARS-CoV-2 infection in patients that received belantamab mafodotin (*p* < 0.001) according to the Fisher’s exact test.

## 4. Discussion

The SARS-CoV-2 pandemic is a major cause of morbidity and mortality worldwide. Patients with MM are at high risk for severe infection, breakthrough infection, and they present suboptimal humoral responses to COVID-19 vaccination [16,17,18,19,20]. Unfortunately, in some patients with cancer, the infection cannot be completely controlled with antiviral drugs and supportive care, and they ultimately develop severe disease and need hospitalization [8]. The majority of immunocompromised patients may not be fully protected from severe infection after vaccination and the early use of oral antiviral drugs in cases of infection seems to be effective in preventing severe COVID-19 [3,5,21,22]. The use of specific antiviral treatment with molnupiravir and ritonavir-boosted nirmatrelvir aims to reduce the risk of severe infection and hospitalization in patients with MM who undergo mild SARS-CoV-2 infection and do not require supplemental oxygen [12,23,24]. Molnupiravir is the prodrug of the ribonucleoside analogue β-D-N4-hydroxycytidine inhibiting the SARS-CoV-2 replication and reducing viral load [23,25,26,27]. In phase I/II/III studies, it has shown both safety and efficacy, reducing the risk of hospitalization and mortality by approximately 50% in non-hospitalized adults with mild-to-moderate SARS-CoV-2 disease who are at risk for poor COVID-19-related outcomes [22]. Ritonavir-nirmatrelvir is an oral protease inhibitor that has also reduced the risk for hospitalization and death by approximately 90% in clinical trials [11]. Βοth molnupiravir and ritonavir-nirmatrelvir are administrated within the first five days of symptoms [12].

Our analysis included patients with MM who were receiving antimyeloma therapy and were infected with SARS-CoV-2. Patients with MM have a high risk of severe infection because of their immunosuppression and their defective response to vaccination [28]. Thus, the patients were eligible to receive either ritonavir-nirmatrelvir or molnupiravir according to the national guidelines. We compared the demographic characteristics of the patients, such as their age, health problems, vaccination status, levels of neutralizing antibodies, antimyeloma therapies, response to therapy, and the severity of their SARS-CoV-2 infection as well as their mortality. No significant differences were revealed between the two antiviral treatment groups in terms of both baseline characteristics and infection outcomes.

No statistically significant difference was observed in the infection outcome regarding the demographic characteristics of the patients including BMI, age, and past medical history defined as the presence of DM, COPD, CAD, or hypertension. Regarding PS, a statistically significant difference of mild cases versus severe cases was observed, while the difference between mild and moderate cases was borderline non-significant. The PS of each patient could be affected by age, BMI, comorbidities, antimyeloma therapy, tolerance to this therapy, and severity of the underlying MM [29]. A longer follow up of our data would enable the characterization of long-term outcomes related to COVID-19, such as the persistence of symptoms related to long COVID.

A difference between severe and mild cases was observed concerning NAbs response levels before SARS-CoV-2 infection. Patients with severe disease had lower neutralizing antibody levels before SARS-CoV-2 infection compared to patients with mild disease. Regarding the role of active treatment, it was observed that treatment with belantamab mafodotin was associated with severe COVID-19 outcomes. Belantamab mafodotin is a humanized IgG1 k monoclonal antibody against the B-cell maturation antigen (BCMA) conjugated with a cytotoxic agent, maleimidocaproyl monomethyl auristatin F (mcMMAF). The antibody–drug conjugate binds to BCMA on myeloma cell surfaces causing cell-cycle arrest and inducing antibody-dependent cellular cytotoxicity [30,31].

Previous studies have consistently shown that patients with MM who receive anti-BCMA therapies, including conjugated monoclonal antibodies and bispecific antibodies, have inferior humoral responses following initial and booster vaccination compared with other treatment regimens [30,31,32]. Pertinent data have also shown that treatments with anti-CD38 monoclonal antibodies or anti-BCMA bispecific T-cell engagers have been associated with inferior CD4+ T-cell responses against SARS-CoV-2as well [5,33]. These patients may be more susceptible to SARS-CoV-2 infection and severe COVID-19. However, it should be noted that only a few patients were receiving anti-BCMA drugs in our study; therefore, the significant results of the univariate analysis should be interpreted with caution. Furthermore, anti-BCMA agents are currently administered in the relapsed/refractory disease setting. The degree of immune dysfunction probably increases with multiple lines of treatment due to exposure to different drug classes and myeloma burden. A larger study sample would be required to perform a robust multivariate analysis and control for potential confounders.

Lasagna et al. collected data from patients with solid tumors on active treatment and examined the effectiveness of oral antivirals in preventing severe SARS-CoV-2 infection and mortality. The majority of treated patients showed a reduction in the duration of symptoms and only one patient required hospitalization. This study confirmed the efficacy of oral antivirals in patients with solid tumors who receive antineoplastic therapy and underlined the need for the early management of patients with cancer who are infected with SARS-CoV-2, in order to avoid both hospitalization and death [6].

Real-world data are important to evaluate both the effectiveness and safety of COVID-19 antivirals in patients with cancer [8]. These data suggest that outpatient therapies for mild SARS-CoV-2 infection may reduce the duration of symptoms, and the risk of hospitalization and morbidity. It seems that the main benefit of the early use of antivirals lies in the prevention of severe infection that requires hospitalization. Our data suggest that the effect of antivirals may be limited when a patient develops severe infection. However, a main limitation of this study pertains to the absence of a placebo or a non-treatment group of patients with myeloma and COVID-19. Therefore, we were not able to precisely determine the benefit of antivirals administration compared to supportive care alone. Furthermore, our study was not randomized in order to minimize any potential bias in the comparisons between the two antivirals. The outcomes may vary depending on the availability of other supportive care, which could be a major factor in determining patients’ disease course. The dominant SARS-CoV-2 variants in the community may also differentiate patient outcomes. During the study period, the Omicron strain prevailed, which has been associated with less severe disease compared to previous strains. A longitudinal analysis during different SARS-CoV-2 waves, including control groups, would be necessary to reach firm conclusions about the precise benefit of antivirals. Although the study sample size was small, our study provides useful real-world data that may be used as a basis for the design of larger randomized studies in the field.

In conclusion, ritonavir-nirmatrelvir and molnupiravir may be beneficial in preventing severe disease from SARS-CoV-2 infection in MM patients who are under anti-myeloma treatment. This prospective study indicated the comparable effects of the two treatment options, providing an important background for further research.

## Figures and Tables

**Table 1:** Patients’ characteristics before SARS-CoV-2 infection.

		Mean	SD
Nabs before SARS-CoV-2 infection (%)		65.77	29.19
		N (N = 169)	%
Performance Status (PS)	0	133	78.7%
	1	36	21.3%
Age	30–50 years	19	11.2%
	50–70 years	96	56.8%
	>70 years	54	32%
Diagnosis	Multiple Myeloma (MM)	160	94.7%
	Smoldering Multiple Myeloma (SMM)	9	5.3%
Line of therapy in MM (N = 160)	1st	100	62.5%
	2nd	36	22.5%
	3rd	14	8.8%
	4th	7	4.4%
	5th	3	1.8%

**Table 2:** Patients’ treatment combinations concerning multiple myeloma: Proteasome inhibitor (PI), immunomodulatory drug (IMiD), SINE (selective inhibitor of nuclear transport).

Regimen	N
PI-based regimen	23 (14.4%)
IMiD-based regimen	43 (26.9%)
PI- and IMiD-based regimen	35 (21.9%)
Anti-CD38-based regimen	38 (23.7%)
Anti-BCMA-based regimen	17 (10.6%)
SINE compound based regimen	3 (1.9%)
Other	1 (0.6%)

**Table 3:** Patients’ responses to the treatment for SARS-CoV-2 infection (hospitalized or non-hospitalized patients).

	Outcome					
	Mild (N = 149)		Moderate (N = 15)		Severe (N = 5)	
	N	%	N	%	N	%
Hospitalization	0	0%	1	6.7%	5	100%
Intubation	0	0%	0	0%	2	40%
Tocilizumab	0	0%	0	0%	5	100%
Corticosteroids	1	0.7%	13	86.7%	5	100%

**Table 4:** Antiviral drug type and outcome of the SARS-CoV-2 infection (M: molnupiravir, R-N: ritonavir-nirmatrelvir).

		Outcome					
		Mild		Moderate		Severe	
		N	%	N	%	N	%
Antiviral Drug	M (N = 30)	27	90%	1	3%	2	7%
	R-N (N = 139)	122	88%	14	10%	3	2%

**Table 5:** Distribution of gender and MM stage according to the outcome of COVID-19 infection.

		Outcome					
		Mild		Moderate		Severe	
		N	%	N	%	N	%
Gender	MALES (N = 95)	82	86.3%	10	10.5%	3	3.2%
	FEMALES (N = 74)	67	90.5%	5	6.8%	2	2.7%
Stage	RISS I (N = 52)	45	86.5%	6	11.6%	1	1.9%
	RISS II (N = 62)	56	90.3%	4	6.5%	2	3.2%
	RISS III (N = 55)	48	87.3%	5	9.1%	2	3.6%

**Table 6:** Differences in COVID-19 outcomes according to medical history, BMI, and age.

	Outcome						
	Mild		Moderate		Severe		
	N	%	N	%	N	%	*p*
Diabetes Mellitus	10	6.7%	3	20%	1	20%	0.099
Copd	15	10.1%	1	6.7%	0	0%	0.697
Cad	6	4%	0	0%	0	0%	1.000
Hypertension	63	42.3%	7	46.7%	1	20%	0.593
	Mean	SD	Mean	SD	Mean	SD	
Bmi	26.80	4.46	28.27	6.76	25.88	3.36	0.456
Age	64.34	10.60	65.00	9.30	65.00	12.29	0.966

**Table 7:** Differences in COVID-19 outcomes depending on performance status, response to treatment for MM, and NAbs before SARS-CoV-2 infection (SD: stable disease, PR: partial response, VGPR: very good partial response, CR: complete response, sCR: stringent complete response).

		Outcome						
		Mild		Moderate		Severe		
		N	%	N	%	N	%	*p*
Performance Status	0	122	81.9%	9	60%	2	40%	0.017
	1	2	18.1%	6	40%	3	60%	
RESPONSE	SD	15	10.5%	5	33.3%	2	40%	0.126
	PR	33	23.1%	1	6.7%	0	0%	
	VGPR	52	36.4%	4	26.7%	2	40%	
	CR	32	22.4%	3	20%	1	20%	
	sCR	11	7.7%	2	13.3%	0	0%	
		Mean	SD	Mean	SD	Mean	SD	
NAbs before COVID (%)		67.49	28.85	59	24.69	35.4	37.57	0.040

## Data Availability

Data are available upon request from the corresponding author.
